# Automatic Fortran to C++ conversion with FABLE

**DOI:** 10.1186/1751-0473-7-5

**Published:** 2012-05-28

**Authors:** Ralf W Grosse-Kunstleve, Thomas C Terwilliger, Nicholas K Sauter, Paul D Adams

**Affiliations:** 1 Lawrence Berkeley National Laboratory, Cyclotron Road, BLDG, 64R0121, Berkeley, CA, 94720-8118, USA; 2 Los Alamos National Laboratory, Los Alamos, NM, 87545, USA; 3 Department of Bioengineering, University of California Berkeley, Berkeley, CA, 94720, USA

**Keywords:** Fortran, C++, Source-to-source conversion, Python, Test-driven development

## Abstract

**Background:**

In scientific computing, Fortran was the dominant implementation language throughout most of the second part of the 20th century. The many tools accumulated during this time have been difficult to integrate with modern software, which is now dominated by object-oriented languages.

**Results:**

Driven by the requirements of a large-scale scientific software project, we have developed a Fortran to C++ source-to-source conversion tool named FABLE. This enables the continued development of new methods even while switching languages. We report the application of FABLE in three major projects and present detailed comparisons of Fortran and C++ runtime performances.

**Conclusions:**

Our experience suggests that most Fortran 77 codes can be converted with an effort that is minor (measured in days) compared to the original development time (often measured in years). With FABLE it is possible to reuse and evolve legacy work in modern object-oriented environments, in a portable and maintainable way. FABLE is available under a nonrestrictive open source license. In FABLE the analysis of the Fortran sources is separated from the generation of the C++ sources. Therefore parts of FABLE could be reused for other target languages.

## Background

The work presented here grew out of the development of a software suite for the determination of macromolecular structures using crystallographic methods [1]. Crystallographic computing has been connected to language development from the earliest days of scientific software when David Sayre, who is mainly known for his contributions to crystallography [2], was also a member of the original Fortran development team [3]. This early influence is still evident in a substantial amount of crystallographic software implemented in Fortran 77 [4]. At the same time, developments in computer science have led to the wide-spread use of object-oriented languages.^[a]^ Integrating time-tested Fortran implementations into modern object-oriented software environments is often problematic (see below), yet in many cases it would be prohibitively expensive to replace existing Fortran implementations with a new object-oriented implementation. This is because scientific algorithms tend to be highly specialized and their understanding usually requires complex domain-specific knowledge. Often only a relatively small fraction of a development effort is spent on coding. If a new developer has to acquire domain-specific knowledge for a rewrite in another language, the project completion time can be very similar to the initial one. This problem worsens over time as the complexity of scientific methods is steadily increasing.

Integrating existing Fortran implementations into object-oriented environments is often problematic because most Fortran programs of significant size make extensive use of global variables, which means that they cannot safely be used as building blocks in modular or multithreaded systems [5]. Another problem is that many Fortran programs rely on the file system for communicating intermediate data between different parts of a workflow. On modern clustered multi-core systems this can easily lead to I/O congestion. Typical Fortran programs can only be used as coarse-grain modules, usually through scripts that create the input, run the program, and harvest the output. Such systems tend to be difficult to install and maintain. Some Fortran libraries, notably LAPACK [6], are written in a style that makes them suitable for fine-grained use in a modular system. However, using Fortran implementations from another programming language significantly reduces portability since the procedures for compilation, linking, and distribution become highly environment specific, in particular on platforms that lack a native Fortran compiler (for example Windows and Mac OS X). This is a hindrance especially for dynamic collaborative projects supporting multiple platforms. In such situations it is often necessary to rebuild the entire package from sources in a variety of environments. For example, our PHENIX package [1] is developed in a number of geographically spread-out research groups and released very frequently with sources. In our experience the mix of Fortran and C++ [7] was the root cause of numerous portability issues for developers and users. In addition we were confronted with the practical inability to use our own C++ libraries [8] from Fortran, because adding Fortran interfaces to a C++ library is involved, difficult to maintain, and tends to compromise the C++ interfaces. The many difficulties of working with a mixed-language Fortran/C++ system motivated us to develop a tool for the automatic conversion of Fortran 77 code to C++, named FABLE.^[b]^ Our goal for FABLE was to generate C++ code suitable for continued development, by being human-readable, and integration into modern modular systems, by avoiding global variables.

We also found it important to generate C++ code similar to the original Fortran code so that it continues to appear familiar to the original authors.

Conceptually FABLE is similar to the F2C program developed two decades ago [9], but the C code generated by F2C was never intended to be human-readable. The F2CPP [10] script helps by automatically rewriting the F2C output using C++ syntax. However, both F2C and F2CPP convert Fortran global variables to C global variables, which has the drawbacks mentioned above. Commercial Fortran-to-C++ conversion services are offered by a number of companies, and although a full listing is beyond the scope of this article, we did engage such services for a predecessor project [11]. One major issue identified in this project was a dependence on laborious manual changes after the automatic Fortran-to-C++ conversion but prior to testing. During the period of manual changes we could not avoid the continued development of the Fortran sources, therefore these new developments later had to be laboriously converted and merged with the C++ code. FABLE avoids this situation by permitting the converted code to be immediately tested. A second major issue was that the C++ code generated in the conversion preserved Fortran global variables as C++ global variables. It was therefore necessary to develop a series of ad-hoc scripts to automatically rewrite the C++ code, moving Fortran global variables to C++ *structs*. The result of these automatic rewrites is similar to the approach now used by FABLE, as described below.

We decided to release FABLE as open source, with the goal of making it attractive for open research projects, particularly when a number of diverse small conversions are needed from time to time, for which repeated use of a commercial service may be impractical.

### Implementation

**Figure 1:**
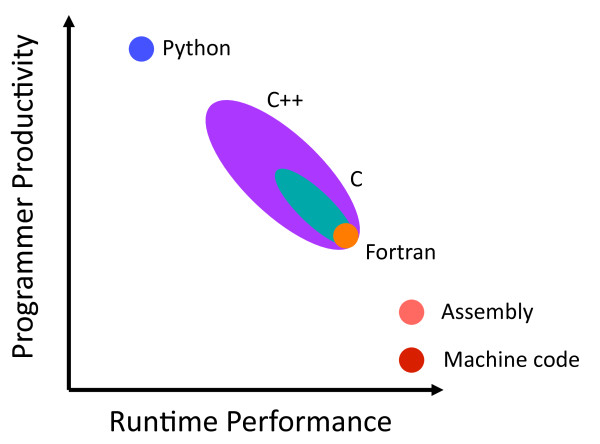
Qualitative graph of runtime performance vs. programmer productivity for selected programming languages.

The Python [12] scripting language was chosen as the implementation language for FABLE. The rationale for this choice is illustrated qualitatively by Figure 1, which reflects two decades of experience working with Fortran, C/C++ and Python, and some limited experience working with assembly and machine code. In our work we observe a clear negative correlation between programmer productivity and the degree to which a programming language emphasizes runtime performance. Implementing new algorithms is most quickly achieved using Python, but Python’s dynamic typing and the fact that it is an interpreted language lead to a relatively low runtime performance. Machine code at the other end of the spectrum is very time-consuming to write but will most likely be the fastest to execute. The other languages fall in between these extremes. We anticipated that the execution times for transforming source code with Python would be sufficiently short to be able to take advantage of this highly productive language. Importantly, compared to other languages, Python scripts are usually very amenable to future developments by new contributors.

The C++ code generated by FABLE depends on the FEM Fortran EMulation library which we developed along with the FABLE code generator. The FEM library in turn depends only on ISO standard C/C++ libraries. During development we used some Boost libraries [13] to accelerate the development process. In the final stages of FABLE development, when it was clear what was actually needed, we removed the dependency on the Boost libraries by writing a small amount of C++ code with minimal replacements.

A test-driven approach was adopted during FABLE and FEM development. For each development step, we generated a small file with Fortran code to be converted, implemented the corresponding new processing steps, and added tests to exercise the new features. As the number of tests grew during development, we parallelized the execution of the tests. In this way it was possible to keep the time for a full test cycle below one minute, using hardware with 48 processor cores. To ensure that existing functionality is not destabilized by new developments, the FABLE tests are automated and run routinely in nightly multi-platform builds (follow links from cctbx.sourceforge.net).

## FABLE design

### Preliminary remark

In the text below we frequently use the term “C++ *struct*”. We note that a “C++ *struct*” is similar to a C++ “*class*”. The difference between a C++ *struct* and a *class* is that full “public” access to members is the default for a *struct*, and restricted “private” access is the default for a *class*. Such access restrictions do not exist in Fortran 77; all global variables can be accessed for reading and writing from any procedure. To reflect this, and to keep the FABLE- generated C++ code simple, we chose to work with *structs*.

### Overall conversion workflow

We had previous experience converting a certain part of the PHENIX Fortran sources using a commercial service [11]. From this work we had learned that the success of a conversion project critically depends on the amount of manual work required before the automatically converted source code can be compiled and tested. For FABLE, our highest priority was therefore to eliminate the need for manual work between automatic conversion and testing.

We had also learned that making minor modifications to the Fortran code prior to conversion can significantly improve the resulting C++ code. Therefore our second major decision was to design FABLE as a tool to be applied iteratively. The third major decision was to make

FABLE open-source so that it can also be a dynamic component in the development process of any conversion project. The conversion workflow then consists of convert-build-test cycles in which both the Fortran code and the FABLE code generator can be modified to automatically obtain the final C++ sources.

The next major design decision was to emulate the Fortran I/O system. This decision had several motivations. The most important was to help with our highest priority, the elimination of manual work between automatic conversion and full testing. The next motivation was to help with the goal of making the generated C++ code similar to the original Fortran code, as stated in the introduction. The third motivation was to provide a foundation for the complete encapsulation of all I/O operations performed by the original Fortran code. For example, opening a new file could be re-directed to initializing a buffer in memory, writing to the file could add to the buffer, and closing the file could trigger any procedure using the buffered output, without ever leading to interactions with the file system. This mechanism is designed to help eliminate I/O bottlenecks on modern machines with a large number of processor cores, without, importantly, having to change the design of the original implementation.

Another major design decision was to fully encapsulate the state of a Fortran program in a single C++ object. For this, all Fortran 77 global (COMMON) and persistent (SAVE) variables are converted to C++ *struct* members. Each common block becomes a C++ *struct*. Another *struct* is generated for each Fortran procedure (PROGRAM, BLOCKDATA, SUBROUTINE, and FUNCTION) containing SAVE variables. All COMMON and SAVE *structs* are combined into one potentially large *cmn* object that also contains the state of the I/O system. In this way an entire Fortran program is rendered a reusable building block for a modular system. We note that multiple *cmn* objects can exist in the same process; under favorable conditions (a thread-safe C++ *new* implementation, with no conflicting I/O) they may even be used concurrently. Each *cmn* object can be made to persist after the (virtual) end of the original Fortran program, for example to extract results directly from arrays. It is also possible to change the state of the *cmn* object and to arbitrarily call the converted Fortran functions. This organization opens an evolutionary path to an object-oriented re-organization after the automatic conversion is finished.

### FABLE implementation

The main components of FABLE are a *read* module and a *cout* code generation module. The *read* module is supported by a *tokenization* module that handles both general fixed-form Fortran 77 syntax and Fortran FORMAT specifications. (A small subset of Fortran 90 extensions is also supported, to enable conversion of LAPACK [6]). Fortran reading and C++ code generation are clearly separated, with the idea that the *read* module can be reused by code generators for other target languages.

The FABLE *read* module builds a call graph of all the input sources. It then performs a topological sort with handling of dependency cycles, followed by an automatic determination of variables that are *const*[7], even tracing through function pointers. FABLE can be directed to extract only a subset of the Fortran procedures (along with all its dependencies). Unused procedures are not converted.

### Major features of FABLE-generated C++ code

Historically Fortran was the first compiled language and it has clearly influenced the generations of languages that followed, including the C/C++ language family. Therefore Fortran assignments and expressions translate directly to C++, except for the power operator. The Fortran control statements are an approximate subset of the C++ control statements, with minor syntactic differences that are straightforward to translate. Fortran procedures correspond directly to C++ functions, and Fortran scalar types can be mapped directly to C++ types, although a few assumptions have to be made. For example FABLE assumes that the Fortran types INTEGER, REAL and DOUBLE PRECISION map to the C++ types *int, float,* and *double*, respectively. On most current computing platforms, the sizes of the C++ types should be identical to the sizes of the corresponding Fortran types. The only exception is the mapping of the Fortran LOGICAL type to the C++ *bool* type. In this case we found it more important to map the concept rather than the implementation detail that a LOGICAL occupies four bytes on most platforms while the size of a C++ *bool* is one byte on most platforms. This small asymmetry has to be kept in mind when calling external libraries from FABLE-generated C++. The Fortran COMPLEX and CHARACTER types are mapped to C++ template classes, *std::complex* in the C++ Standard Template Library [7] and *fem::str* in the FEM library, respectively. The storage patterns in memory are identical to Fortran in both cases. More details about the mappings from Fortran types to C++ types can be found in the FABLE documentation.

In the following subsections we highlight other selected features of FABLE-generated C++ code and present the underlying rationale. We believe that the approaches below should work for all conversion projects, but note again that the code generator is openly available and could be customized for unusual situations.

### Array types

A major shortcoming of C/C++ compared to Fortran 77 is the absence of an intuitive multi-dimensional array type. The syntax for built-in C/C++ multi-dimensional arrays is more verbose (requiring a series of square-bracket pairs), the origin for each dimension is fixed at zero, and when passing multi-dimensional array references the sizes of the fast dimensions have to be compile-time constants. This alone makes C/C++ built-in arrays unsuitable as a substitute for Fortran 77 arrays, but in addition the Fortran storage order in memory is transposed compared to the storage order of C/C++ built-in arrays. Many existing Fortran algorithms depend critically on the storage order and would have to be rewritten in major ways to work with the C/C++ storage order. We believe such intricate algorithmic changes will be beyond the reach of automatic conversion tools in the foreseeable future; but we also believe that such changes have little or no practical value, except that it is more difficult for software developers to switch between two conventions. We decided to accept this drawback and emulate Fortran 77 multi-dimensional arrays using the object-oriented features of C++. This decision is also motivated by the previously stated goal of making the generated C++ code similar to the original Fortran code.

**Figure 2:**
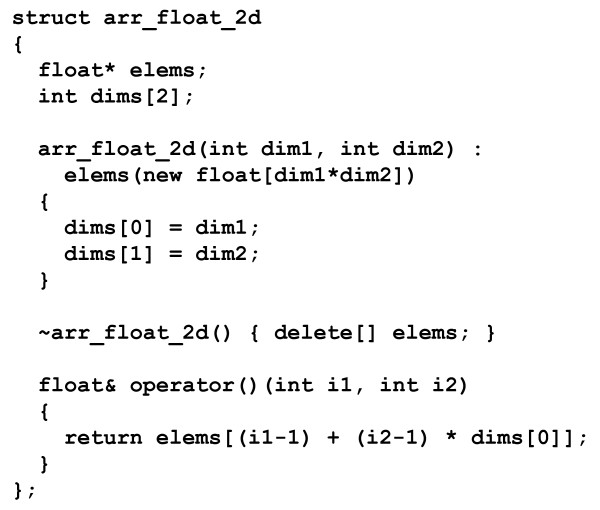
**Essence of Fortran multi-dimensional array emulation in C++** We note that the array type is noncopyable in the actual implementation, since this both emulates Fortran behavior and is a simple way to ensure matching new/delete calls.

The principle for emulating Fortran arrays in C++ is very simple. The full implementation in the FEM library, which includes comprehensive handling of DIMENSION statements, is approximately 1,500 lines of C++, but a compilable essence is contained in less than twenty lines in Figure 2. The example *struct* manages a pointer (*elems*) to a dynamically allocated block of memory for the array elements and also stores the sizes of the two dimensions; for simplicity the origin is fixed at one. The C++ constructor of the example *struct* allocates the memory, the destructor frees it, and the call-operator performs the two-dimensional index calculation to obtain a reference to a particular array element. In the FEM library the array element type and the number of dimensions are C++ template parameters. An overview of the full family of FEM C++ array and dimensioning types can be found in the FABLE documentation.

### Common and save variables

Fortran common blocks are used to share data between procedures, but Fortran 77 lacks features that formally associate common blocks with particular procedures. All common variables are global to the entire program. A possible mapping of a Fortran common block to C++ would be to introduce a C++ namespace (with the name of the common block) that contains the variables. However, this does not solve the problem of having global variables [5]. Therefore FABLE maps a common block to a C++ *struct*, an approach introduced in [11]. This is a first step towards an object-oriented re-organization of the converted code. If the common *structs* are closely associated with certain procedures, these procedures can manually be turned into C++ member functions to formalize the dependencies. C++ inheritance is used to combine all common *structs*, by adding them as base classes to the *cmn struct*. Variables can then be accessed via *cmn.varname*. This approach leads to concise and obvious code in most practical cases, but has the problem of potential naming conflicts. Fortran common blocks that are only used in disjoint sets of procedures are joined by FABLE into the C++ *cmn* object. If the same variable name is used for members of two distinct common blocks, in two different procedures, access in C++ must be disambiguated. FABLE solves this problem by analyzing naming conflicts and selectively inserting C++ up-casts from the *cmn* object to specific common *structs.*^[c]^

**Figure 3:**
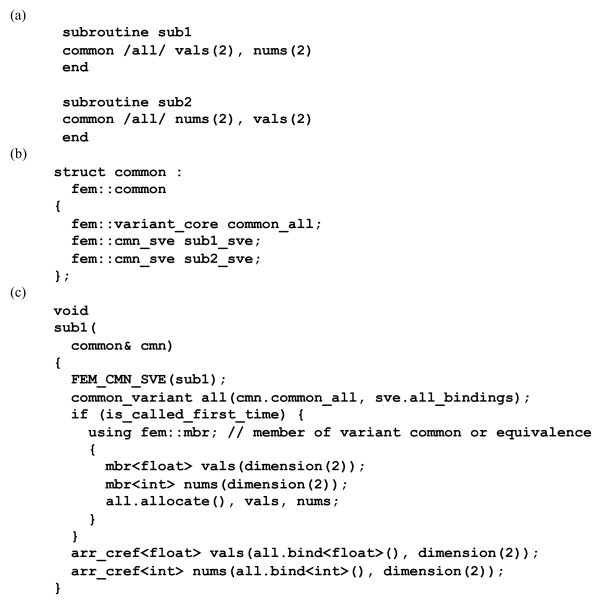
**Example illustrating the handling of common variants by emulating the Fortran compile-time allocation mechanism at runtime in C++.** (a) Two Fortran subroutines with variant uses of common block *all*. (b) Fragment from FABLE-generated C++ code adding placeholders for the *all* common variant and bookkeeping variables for the two subroutines to the *common* struct (which is the type of the *cmn* object). (c) Fragment from FABLE-generated C++ code defining function *sub1*. The *FEM_CMN_SVE* macro defines the *is_called_first_time* and *sve* variables. The latter manages both original Fortran *SAVE* variables and the function-specific bookkeeping for common variants. The *mbr* objects are temporary objects collecting information about variable types and array sizes. This information is used by *all.allocate* to allocate the required memory. The *all.bind* calls define array references to the memory area of the common block for local use in the function. (The FEM library guards against improper allocations that could invalidate existing references in other functions.) The common-variant mechanism shown in the example is meant to provide a general approach for obtaining a first working C++ version of a program. The disproportional length of the generated C++ code in the example highlights why it can be important to incrementally modify the Fortran sources in convert-build-test cycles, as described in section 3.1.

Certain common blocks cannot be mapped to C++ *structs*. Many existing Fortran programs, including the programs we converted for PHENIX, reuse some common blocks for variables of different types and array sizes. We call such situations *common variants*. The motivation for common variants goes back to times of more limited memory resources and the lack of dynamic memory allocation in Fortran 77. It is, in general, difficult to re-organize the Fortran code to avoid common variants; the danger of accidentally introducing subtle critical errors is large. Another consideration is that C++ offers far more versatile tools for re-organizing the code after the automatic conversion is finished. Therefore we decided to emulate the Fortran compile-time allocation of common variants in C++ at runtime, with support code added to the FEM library. A small example is shown in Figure 3. The advantage is that the Fortran code can be automatically converted and tested as-is. A disadvantage apparent from Figure 3 is the relatively large volume of generated C++ code. However, if the C++ code is further developed in the future, it is straightforward to remove the code related to common variants and to use dynamically allocated C++ container types in its place.

SAVE variables are local to a procedure, but persist between calls. Based on considerations similar to those outlined for common variables, for each Fortran procedure with save variables, FABLE generates a C++ *struct* with the variables as the members. The save *structs* are added to the *cmn* object via proxy objects similar to *boost::any*[13]. Save variables are allocated and initialized the first time the associated procedure is called. The indirection through proxy objects makes the *cmn* object independent of the save *structs*. The save *struct* definitions can therefore be kept in separate files, together with the corresponding C++ function definitions. This organization improves readability of the C++ code and was found to greatly reduce compile times for large projects.

### Equivalence statements

Some of the Fortran codes in our sphere of interest make pervasive use of EQUIVALENCE statements. Many uses could be mapped to simple C++ references, but equivalences can also lead to rather complex situations. For example, equivalences can increase the size of a COMMON block. Changing the Fortran code to remove equivalences can be very difficult and also carries a high risk of introducing subtle critical errors. Therefore we took the approach of automatically covering all features of equivalences, including equivalences with mixed types and equivalences that change the size of a common block. This approach is related to our handling of common variants and has the same drawback in that the corresponding volume of generated C++ code is relatively large. However, most importantly, the generated C++ code can be compiled and fully tested. FABLE can be directed to generate more compact C++ code making simplifying assumptions under user direction. The simplifying assumptions can then be validated by retesting. Equivalences involving SAVE and local variables are handled by reusing the tools developed for COMMON equivalences. However, support for applying simplifying assumptions in these cases is not implemented, mainly because the reduction in generated C++ code size would be minor in the projects we have worked on.

### Input/output statements

Emulation of the Fortran 77 Input/Output system constitutes the largest part of the FEM library. The I/O emulation localizes the effort required to convert uses of the Fortran I/O system. The alternative would be to use the C++ I/O system directly and to have a looser correspondence between original Fortran code and generated C++ code. In our experience [11] this is far more difficult to achieve automatically and the generated code lends itself less to continued development. Furthermore, while the I/O emulation is the largest part of the FEM library, it is small compared to the standard C/C++ libraries. The compilation and maintenance overhead associated with the I/O emulation is therefore relatively minor. Another advantage of localizing the I/O activity is the potential for full encapsulation as outlined before.

**Figure 4:**
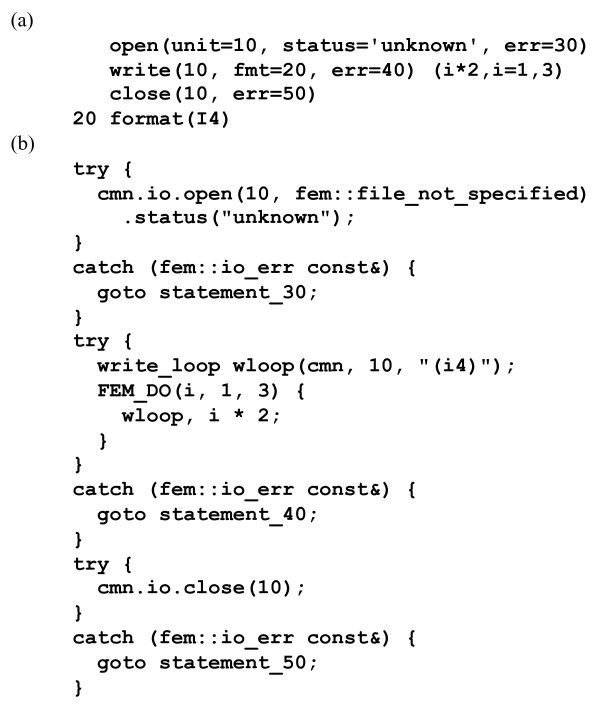
**Example illustrating the design of the*FEM*I/O emulation.(a)** Fortran code. **(b)** C++ code. Each Fortran statement is converted to a C++ building block. The size of the C++ code is relatively large due to the *err* labels. Most *write* statements in real code do not make use of *err* labels, in which case the corresponding C++ code is nearly identical to the original Fortran code.

The I/O emulation makes it possible to map each of the Fortran I/O statements independently to a building block of C++ code. Figure 4 shows an instructive example. The most difficult Fortran constructs to convert are implied-do lists, since there is no direct correspondence in C++. FABLE solves this problem via C++ objects implemented in the FEM library that manage the state of an implied-do list. These *read_loop* and *write_loop* objects appear in regular loops as shown in Figure 4. FABLE can convert arbitrarily deeply nested implied-do lists. Fortran *end* and *err* labels are handled via the C++ exception handling system (*try* and *catch*).

### DATA statements

Scientific source codes tend to include significantly large sections with Fortran DATA statements, for example to store tables with reference data. Mapping DATA statements to C++ is complicated by three features unique to Fortran: the item repeat syntax, implied-dolists, and mixed variable types. FABLE, with support from the FEM library, can convert any Fortran 77 DATA statement to C++. However, to support item repeat counts and item lists with mixed variable types, all C++ items have to be objects similar to *boost::any*[13]. This leads to relatively long compile times and increased sizes of machine code. To simplify the generated C++ code if possible, FABLE analyzes each DATA statement. If a particular statement does not use the item repeat syntax and if all items are of the same type, FABLE generates C++ code using basic array initialization mechanisms. DATA implied-do lists are converted in the same way as I/O implied-do lists, using explicit C++ loops and a C++ object in the FEM library that manages the state of each loop.

### Dynamic parameters

The lack of dynamic memory allocation in Fortran 77 forced many authors to code array size limits into their programs. Users of Fortran 77 programs will be familiar with error messages instructing them to change the source code and recompile. In most cases the size limits are coded as Fortran PARAMETER statements. FABLE can be directed to automatically replace parameters of a certain name with dynamic values that can be changed from the command line. For simplicity, the parameter name is assumed to be global to the program. For each parameter name specified by the user, FABLE adds a variable to the *cmn* object, which is then used as a replacement for the original Fortran constant in all related C++ functions. FABLE also generates the C++ code for inspecting the command line. Integer options are assigned to the dynamic parameter variables in the order given. Therefore, instead of having to recompile, users or higher-level automatic procedures can simply call the converted program with different arguments.

## Results and discussion

Our initial goal was to convert the program SOLVE [14]. The SOLVE Fortran code consists of about 83,000 lines total, with about 61,000 non-empty, non-comment lines. The type of most SOLVE variables is declared implicitly. SOLVE makes use of 86 C functions from the CCP4 library [15]. Most of the SOLVE-specific conversion effort was spent on handling these external library calls. We wrote a set of Fortran “stub” procedures with empty bodies as substitutes for the external procedures. These enabled FABLE to derive the correct *const* information and generate compilable C++ code. The next step was to direct FABLE to write the C++ functions corresponding to the library calls to separate files. These files were modified manually to insert calls to the C library functions. At this stage the C++ version could be compiled, linked against the CCP4 library, and fully tested with an existing suite of 32 tests. The initial size of the generated C++ code was about 180,000 lines. A large portion of the generated code was due to the fully-general default handling of COMMON equivalences. We applied the incremental approach outlined above, directing FABLE to assume simple equivalences (that do not increase the size of the common block) for certain common blocks. This work was guided by conversion reports produced by FABLE. Each trial cycle consisted of re-converting, compiling, linking, and testing, which could be completed in about two minutes on current hardware with 48 processor cores. We made a limited number of changes to the Fortran code that allowed FABLE to generate more compact C++ code. Each of the changes was validated with the test suite. The final size of the C++ code is 127,000 lines, including all comment lines automatically transferred from the Fortran code. The increased size compared to the Fortran code is partially due to the wide- spread use of implicit variable declarations in Fortran, which must appear explicitly in the C++ code; for clarity those declarations appear on separate lines, one for each variable. For completeness we mention that the generated C++ code was automatically split into several files, which necessitated generating an additional header file with about 2,600 lines of C++ function declarations. The C++ version of SOLVE has been in production use in PHENIX since July 2010. The final Fortran code used in the conversion is still available for reference.

Current C++ compilers produce an executable that is about 30–40% slower than the corresponding best available Fortran executable on the same machine (see also the following section). However, as a by-product of the conversion we found a few simple optimization opportunities, which were implemented in the Fortran version. The net result is that the final C++ version is generally as fast as the initial Fortran version. The final Fortran code and the Python scripts directing the conversion with FABLE are included in the PHENIX distributions, available at http://phenix-online.org. The main FABLE conversion script is solve_resolve/solve/run_fable.py, supported by the utility script solve_resolve/fable_utils.py.

 Our second goal was to convert selected algorithms from the LAPACK library (version 3.2.1, [6]). FABLE generates C++ code for the entire library, but a very small subset of the converted C++ code cannot be compiled, due to the use of function pointers in the Fortran code for which there is no caller; FABLE needs at least one caller to generate compilable C++ code for such Fortran code. As we did not have an interest in using this subset, we used the automatic dependency analysis of FABLE to extract only certain eigenvalue (DSYEV) and SVD (DGESVD, DGESDD) algorithms.^[d]^ The size of the selected Fortran code is 26,876 lines. The size of the corresponding C++ code including all comments is 29,592 lines. The moderate 10% increase reflects the absence of COMMON and EQUIVALENCE statements in the LAPACK sources and that all LAPACK Fortran variables are declared explicitly. Systematic runtime comparisons are shown in the following section. A standalone package with the selected Fortran sources and the converted C++ sources, each combined into a single file for easy compilation, are available at http://cci.lbl.gov/lapack_fem/. The package also includes the small shell script that was used for the conversion.

The third major FABLE-based project was the conversion of parts of the MOSFLM program for processing of raw crystallographic data [16-18]. The MOSFLM Fortran sources comprise about 268,000 lines total, with about 126,000 non-empty, non-comment lines. Like SOLVE, MOSFLM makes use of the CCP4 library. Additionally it links to C interfaces of a windowing system and a C library with crystallographic indexing algorithms [18,19]. Our interest is restricted to the computational core of MOSFLM, which we wish to eventually use through Python extension modules, for incorporation into LABELIT [20] and PHENIX. Our first step towards this goal was to reduce the dependencies. For this we needed to analyze which parts of the MOSFLM sources are involved in the computational core of interest. We approached this empirically by developing a set of tests exercising all the functionality needed for our purposes (knowing that these tests would be useful again later for validating the generated C++ code). Then we used FABLE to automatically insert write statements as the first statement into all Fortran procedures.^[e]^After running all tests we harvested the names of all the procedures called during their execution from the output of these write statements. Then we reverted to the original Fortran sources, without the additional write statements. FABLE was then instructed not to convert the bodies of the unused procedures. The remainder of the conversion project was similar to the SOLVE conversion described before, except that we needed to supply stub procedures not only for the CCP4 library calls, but also for calls into the additional C libraries. Since the MOSFLM conversion was the third major FABLE application, we needed to make only very few adjustments to the FABLE C++ code generator. The size of the generated C++ code is about 185,000 lines total; the execution time for the automatic conversion with FABLE is about 54 s, using Python 2.7.1 and one AMD 6174 core (2.2 GHz clock speed) under Fedora 13 Linux. The total effort required for reaching our milestone where the C++ sources passed all tests was approximately three person weeks; this includes the time spent developing the tests. For comparison we note that MOSFLM is the result of over 25 years of development, involving several people. We are planning to continue our MOSFLM related developments by refactoring the current standalone C++ executable as a Python extension module. The Python scripts used in the conversion are available upon request. The scripts are similar to the ones used in the SOLVE conversion.

### Runtime comparisons

Table 1 shows absolute and relative runtimes of the LAPACK DSYEV procedure (see previous section) and a simplified structure factor calculation implementation as introduced in [11], which can also be found in the FABLE source tree (fable/test/sf_times.py). The DSYEV procedure was chosen as an example of a typical linear algebra algorithm that spends very little time in math library functions. In contrast, the structure factor calculation is dominated by calls to math library functions, namely the *exp*(), *sin*(), and *cos*() functions. The structure factor calculation was implemented with both double precision and single precision floating-point arithmetic. All test programs were compiled and run on the same machine (Intel® Xeon® CPU X7350 @ 2.93 GHz, 4 × 4 cores, running the 64-bit version of the Fedora 8 operating system) using various versions of the Intel® Fortran and C++ compilers (software.intel.com), the gfortran and g++ compilers in the GNU Compiler Collection (GCC, gcc.gnu.org), and a recent development version of the CLANG C++ compiler (clang.llvm.org). The –O optimizer option was used for all Intel® compilers. The –O3 –ffast- math options were used for all GCC compilers and CLANG. In addition, the –march=native option was added for these compilers if available. We note that the optimizer options enable auto-vectorization for all compilers that support this feature. Each executable was run eight times. The shortest runtime is reported in Table 1. For compilers that support the –march = native option, the shortest runtime of eight runs with this option and eight runs without is reported. All runs except those reported in the last row of Table 1 made use of the Intel® C++ Version 11.1 math libraries (by setting the LD_PRELOAD environment variable; for further details inspect the fable/test/sf_times.py script).

**Table 1:** Systematic runtime comparisons

	**LAPACK 3.2.1 DSYEV double precision**		**Structure factors double precision**		**Structure factors single precision**	
	**Fortran**	**C++**	**Fortran**	**C++**	**Fortran**	**C++**
**Intel 12.1**	**1.00**	**2.51**	**1.00**	**0.99**	**1.00**	**0.97**
	1.82 s	4.56 s	2.09 s	2.07 s	1.55 s	1.51 s
**Intel 11.1**	**1.03**	**2.30**	**1.01**	**1.01**	**1.09**	**1.06**
	1.88 s	4.18 s	2.12 s	2.11 s	1.69 s	1.65 s
**Intel 10.1**	not	**1.43**	not	**1.06**	not	**1.05**
	installed	2.60 s	installed	2.21 s	installed	1.62 s
**Intel 9.1**	**1.32**	**1.75**	**1.07**	**1.19**	**1.03**	**1.17**
	2.41 s	3.19 s	2.24 s	2.49 s	1.60 s	1.82 s
**gcc 4.6.1**	**1.21**	**1.68**	**1.00**	**1.02**	**1.04**	**1.05**
Intel math library	2.20 s	3.05 s	2.10 s	2.14 s	1.61 s	1.63 s
**gcc 4.5.3**	**1.25**	**1.42**	**1.56**	**1.00**	**1.70**	**1.05**
Intel math library	2.27 s	2.59 s	3.27 s	2.10 s	2.63 s	1.63 s
**gcc 4.4.6**	**1.19**	**1.56**	**1.54**	**1.00**	**1.67**	**1.04**
Intel math library	2.17 s	2.84 s	3.22 s	2.10 s	2.59 s	1.61 s
**gcc 4.3.6**	**1.22**	**1.36**	**1.61**	**1.07**	**1.72**	**1.10**
Intel math library	2.22 s	2.48 s	3.37 s	2.24 s	2.67 s	1.70 s
**gcc 4.2.4**	**1.35**	**1.46**	**1.61**	**1.21**	**1.74**	**1.39**
Intel math library	2.45 s	2.65 s	3.37 s	2.52 s	2.69 s	2.15 s
**gcc 4.1.2**	**1.33**	**1.50**	**1.61**	**1.22**	**1.75**	**1.30**
Intel math library	2.42 s	2.73 s	3.37 s	2.55 s	2.71 s	2.01 s
**clang 3.1 r142719**	not	**1.37**	not	**1.09**	not	**1.27**
Intel math library	applicable	2.49 s	applicable	2.27 s	applicable	1.97 s
**clang 3.1 r142719**	not	**1.40**	not	**3.03**	not	**10.19**
System math library	applicable	2.55 s	applicable	6.34 s	applicable	15.79 s

Systematic runtime comparisons using various Intel® and GCC Fortran and C++ compilers, and a recent development version of CLANG++ (LLVM/CLANG SVN revision 142719). Runtimes relative to that of executables compiled with Intel® Fortran Version 12.1 are shown in bold. The corresponding absolute runtimes in seconds are shown below the bold numbers. See text for further details.

We observe that the runtime performance of the Intel® Fortran Version 12.1 executable is generally the best or nearly the best. To ease the interpretation of the runtimes shown in Table 1, the numbers shown in bold are the runtimes relative to the Intel® Fortran Version 12.1 reference executable. The DSYEV executable obtained with the GCC 4.3.6.

C++ compiler is the fastest C++ executable. It is 36% slower than the Intel® Fortran reference executable. This is similar to our observations reported in the previous section. Somewhat surprisingly, the Intel® C++ Version 11.1 and 12.1 executables show outstandingly poor performance, while the performance of the Intel® C++ Version 9.1 and 10.1 executables is more comparable to that of the GCC and CLANG executables. In our experience, such surprises are rather common. A particular algorithm may perform surprisingly well or poorly when compiled with a particular compiler or particular optimizer options. If this is a concern, small manual code optimizations can make a significant difference for a particular set of compilers. (We did not have a sufficiently strong motivation to attempt this for the DSYEV implementation.)

The structure factor calculation C++ executables using the Intel® math libraries are mostly nearly as fast as the Fortran reference executables, or even marginally faster. It is striking to see that older g++ executables clearly outperform the corresponding gfortran executables. The g++/gfortran performances are on par only with the latest GCC release (4.6.2). The relative runtimes comparing pairs of double precision versus single precision executables are generally very similar.

The last row of Table 1 was added to highlight the importance of the math libraries used.

Instead of the Intel® math libraries used in all other cases, the system math libraries are used, in combination with the same CLANG executable used in the second-to-last row of Table 1.

A comparison of the last two rows in Table 1 shows that the DSYEV performance is hardly affected by the choice of math libraries, but the structure factor calculation runtimes increase by a factor of 2.8 for the double precision version and even 8.0 for the single precision version. Detailed inspection [11] revealed that this is mostly due to the particularly poor performance of the single-precision *exp*() function in the system math libraries.

### Outline of unit tests

The system of FABLE unit tests can be best understood by considering the way it was built up. We expect that an understanding of the incremental development and testing approach will help guide users seeking to adapt FABLE for their purposes.

The first major step developing FABLE was the implementation of a *read* module (tokenizer and parser) that builds an internal representation of the Fortran sources. The development was strictly test-driven. Based on informal reviews of the target Fortran codes, small Fortran fragments were added as separate files in the fable/test/valid directory. For each file added, the FABLE *read* and *tokenization* modules were developed to support the required Fortran features. The process of identifying new Fortran features to implement, distilling them into a new file in fable/test/valid, and implementing the required support in the *read* module was repeated many times until all target Fortran codes could be read successfully. The fable/tst_read.py script automatically exercises reading of all Fortran sources in fable/test/valid. In addition, the script systematically exercises the FABLE error diagnostics, covering syntax errors (Fortran sources in fable/test/syntax_error), semantic errors (fable/test/semantic_error), and unsupported Fortran features (fable/test/unsupported).

The second major step developing FABLE was the implementation of the *cout* code generator, simultaneously with the development of the C++ FEM library. The basic approach was very similar to that used in the development of the reader, incrementally implementing new *cout* features targeting a particular file in fable/test/valid; this includes files added while developing the *read* module and additional files to specifically exercise the *cout* module. The generated C++ code is exercised at two levels. The fable/tst_cout.py script systematically exercises the generated code at the text level, with complete test coverage of the *cout* module. This script finishes very quickly (< 1 s) and can therefore be rerun very frequently during development work, for example when refactoring the *cout* module. The fable/tst_cout_compile.py script exercises.

 C++ code generation, automatic compilation and linking with a C++ compiler (e.g. GCC, Visual C++, or CLANG), and running the resulting executable. The script can also be directed to compile the original Fortran code with a Fortran compiler. The output produced by the C++ or Fortran executables is compared with expected output tabulated in the file test/valid/file_names_and_expected_cout. This ensures that the numerical results and the emulation of the Fortran text-format I/O are fully compatible with the results from a particular Fortran compiler. We chose to use the results obtained with the Intel® Fortran compiler as a reference (all Intel® Fortran versions shown in Table 1 lead to identical results processing our test suite; we note that the text-format I/O results using the gfortran compiler are not fully compatible). The internal consistency of Fortran binary-format I/O is also exercised, through binary-format write-read cycles. For completeness we note that we did not have an interest in emulating a particular binary file format and chose to implement our own binary format for simplicity.

The fable/tst_cout_compile.py script can be directed to run the test executables with the valgrind tool (valgrind.org). Valgrind detects many memory access problems, for example uninitialized reads or writes past the end of an array. This was found to be invaluable for guarding against problems in the FEM library. Another highly important feature of the fable/tst_cout_compile.py script is the option to run the tests in parallel on multi-core machines, with the additional option to use precompiled header files. This greatly reduces the time for running the script, for example from 267 to 14 s on current hardware using 48 processor cores. It is therefore possible to re-test frequently during development, which enables a highly dynamic but safe development approach.

## Conclusions

Our initial motivation for developing FABLE was to enable the conversion of the SOLVE program, but the FABLE implementation is designed to be reusable and extensible. The additional work required to also enable automatic conversion of the bulk of LAPACK and the computational core of MOSFLM was minimal. Our experience with the MOSFLM conversion suggests that most Fortran 77 codes can be converted with an effort that is minor (measured in days) compared to the original development time (measured in years). Handling calls to external libraries for which no Fortran source is available is likely to be the most time-consuming part of a conversion effort. FABLE could be further automated to ease dealing with external library calls and equivalences. We also note that the emulations in the FEM library may have to be further completed for specific projects. To allow the scientific community to participate in future developments, FABLE is released under a nonrestrictive open source license. A comprehensive set of unit tests ensures that FABLE and the FEM library can be improved without destabilizing existing functionality.

With current compilers, the runtime of C++ executables is typically 30–55% longer than that of corresponding Fortran executables. Algorithms that spend a significant fraction of the runtime in the math libraries are less affected, provided that the same math libraries are used. The results in Table 1 indicate that there is still a significant potential for improvement in C++ optimizer technology, because the structure factor calculation C++ executable performance with recent compilers is nearly as fast as the best Fortran performance, but the DSYEV C++ performance still lags behind the Fortran performance. However, our applications suggest that already with current C++ compilers the performance difference can be inconsequential in practical situations. Additionally, modern tools can enable performance gains that are much more difficult to realize in Fortran, for example through hash-based lookups or by building object hierarchies that cache complex intermediate results. Of course, there are many more motivations to work with object-oriented languages. With FABLE it is possible to reuse and evolve legacy work in modern object-oriented environments, in a portable and maintainable way.

## **Endnotes**

^a^ See for example http://langpop.com/.

^b^ The name “FABLE” is short for “Fortran ABLEitung”. “Ableitung” is a german word which can mean both “derivative” and “branching off”.

^c^ The output of the command fable.cout fable/test/valid/common_name_clash.f illustrates how C++ up-casts are used to resolve naming conflicts, for example static_cast < common_cmn1& > (cmn).num2 and static_cast < common_cmn2& > (cmn).num2.

^d^ Using the fable.cout --top-procedure option. The full command can be found in the FABLE documentation (file cout_selected.csh under http://cci.lbl.gov/fable/sources/lapack_fem/).

^e^ Using the command fable.insert_write_at_start_of_each_procedure.

## **Availability and requirements**

**Project name:** FABLE

**Project home page:**http://cci.lbl.gov/fable/ (public SVN hosted under http://cctbx.sourceforge.net/ as part of the CCTBX [8] project)

**Operating systems:** Platform independent

**Programming languages**: Python, C++

**Other requirements:** Python version > = 2.3 and < 3

**License:** CCTBX license (nonrestrictive open source)

**Any restrictions to use by non-academics:** None

## Competing interests

The authors declare that they have no competing interests.

## Authors’ contributions

RWGK developed the design, implementation, unit tests, documentation, and automated multi-platform tests for FABLE. TCT developed the SOLVE tests and adjusted the SOLVE Fortran code to aid the conversion. NKS developed the MOSFLM tests and FABLE Python scripts directing the conversion. PDA proposed the SOLVE conversion, secured the funding, and directed the project. All authors read and approved the final manuscript.
